# Editorial: Therapeutic Advances in Melatonin Research

**DOI:** 10.3389/fphar.2022.947117

**Published:** 2022-06-14

**Authors:** Francisco López-Muñoz, Russel J. Reiter, Javier Egea, Alejandro Romero

**Affiliations:** ^1^ Faculty of Health, University Camilo José Cela, Madrid, Spain; ^2^ Neuropsychopharmacology Unit, Hospital Doce de Octubre Research Institute (i+12), Madrid, Spain; ^3^ UTexas Health San Antonio, Long School of Medicine, San Antonio, TX, United States; ^4^ Molecular Neuroinflammation and Neuronal Plasticity Research Laboratory, Research Unit, Hospital Universitario Santa Cristina, Instituto de Investigación Sanitaria-Hospital Universitario de la Princesa, Madrid, Spain; ^5^ Department of Pharmacology and Toxicology, Faculty of Veterinary Medicine, Complutense University of Madrid, Madrid, Spain

**Keywords:** melatonin, pinealism, cancer, cardioprotection, bipolar depression, molecular

Many research groups worldwide are applying the therapeutic properties of melatonin to a large number of pathologies. Its ubiquitous presence in the body and wide safety margin, as well as its ability to modulate several physiological processes, make it an interesting molecule to be evaluated in prevalent pathologies such as cancer, neurodegenerative disorders, stroke, etc.

Regarding the experimental clinical use of melatonin, it is important to highlight that this indoleamine is not only a well-known anti-inflammatory, anti-oxidative and immune-enhancing agent but also its small size and amphiphilic nature allows the molecule to easily diffuse through any membrane, reaching cytosolic, mitochondrial and nuclear compartment. In this regard, even at high doses, serious adverse side-effects have not been reported; therefore, the therapeutic use of high doses of melatonin as an adjuvant treatment in wide variety of diseases, could be of critical importance for the development of novel therapeutic strategies.

This Research Topic contains 5 papers, including 3 original research articles, 1 systematic review and 1 opinion article. All of them provide important data related the therapeutic value of melatonin for prevention and treatment of depressive disorders, osteoporosis, cardiovascular diseases or its use as an adjuvant in cancer patients.

To explain the disruptions in rhythms associated with bipolar disorder; variations in the biological timing system, instability or phase variations of biological rhythms have been considered. It is known that circadian rhythm of melatonin release becomes irregular with the age and it is observed to deteriorate not only due to physiological conditions but also due to several clinical diseases including mood disorders (MDD). The clock gene disfunction is involved in mood disorders, including seasonal affective disorder, unipolar depression, bipolar disorder and depression vulnerability. In this sense, the use of agomelatine, a specific agonist of MT1 and MT2 melatonergic receptors, has shown efficacy by resetting the disturbed circadian rhythms seen in patients with MDD. Dmitrzak-Weglarz et al. propose in their research article that melatonin is involved in modulating the immune system for unipolar and bipolar depression in two models, i.e., when agomelatine was given to depressed patients (clinical model) and after mouse primary hippocampal neuron cultures were treated with melatonin. The fact that melatonin levels usually declines gradually with age and seasonality may also represent a heritable phenomenon associated with MDD. For the first time, Kunz et al. provided evidence that seasonality of behavior in humans depends on the functioning of the pineal gland.

As above mentioned, melatonin is a powerful antioxidant exerting a multi-organ protective role in many pathological conditions. In this context, to ensure an adequate clinical use of melatonin, the route and timing regimen of administration should be considered. The cardioprotective function of melatonin in humans was evaluated in a systematic review by Dominguez-Rodriguez et al. showing that melatonin administration attenuated heart dysfunction with a favorable effect on the left ventricular ejection fraction.

The potential benefits of melatonin in bone homeostasis, stimulating osteoblastogenesis, and inhibiting osteoclastogenesis, may play a noteworthy therapeutic role for reducing osteoporosis, the most common bone disease. In this regard, Cao et al. reported that melatonin stimulates the osteoblastogenesis promoting an increase of intracellular Ca^2+^ through the STIM1/ORAI1 signalling pathway. This study provides another important evidence for the clinical use of melatonin in the treatment of patients with osteoporosis.

Finally, Egea et al. in their opinion article discuss the remarkable potential of melatonin as candidate for the prevention and supportive treatment of cancer. The adjuvant use of this indoleamine enhances drug efficacies and mitigates chemotherapeutic drug induced-toxicity in cancer patients.

Expectantly, the reader will find in this Research Topic interesting pieces of work that provide evidence regarding the significant utility of melatonin as a versatile regulator that takes part of a large number of neuroendocrine and physiological processes. In view of these observations, and considering melatonin’s low toxicity and its high efficacy, there is obviously a pressing need to design more ambitious preclinical and clinical studies to determine the efficiency of melatonin therapy in a variety of diseases.

